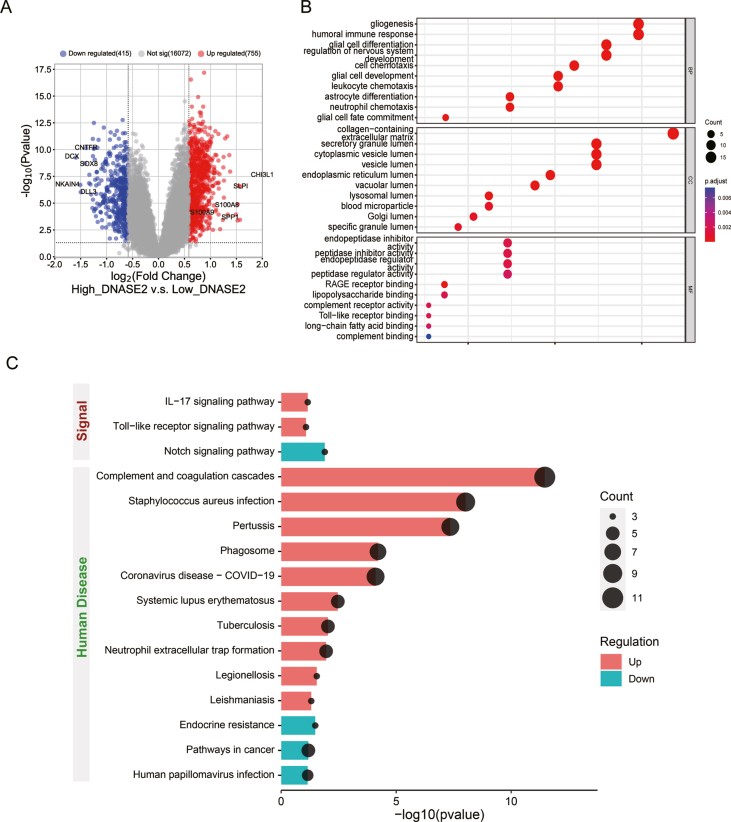# Systematic pan-cancer analysis identifies DNASE2 as a potential prognostic marker and immunotherapeutic target for glioblastoma multiforme

**DOI:** 10.1016/j.gendis.2024.101431

**Published:** 2024-09-10

**Authors:** Jun Cao, Si Chen, Ping An, Xingqiang Wang, Xinru Xiao, Shichao Li, Ye Cheng

**Affiliations:** a Department of Neurosurgery, Xuanwu Hospital, Capital Medical University, Beijing 100053, China; b Department of Neurosurgery, Affiliated Ri Zhao People's Hospital, Jining Medical College, Rizhao, Shandong 276826, China; c Department of Vasculocardiology, Affiliated Ri Zhao People's Hospital, Jining Medical College, Rizhao, Shandong 276826, China; d Experimental Diagnostic Center for Infectious Diseases, The Second Hospital of Hebei Medical University, Shijiazhuang, Hebei 050000, China

Deoxyribonuclease 2 (DNASE2) is associated with tumor proliferation and apoptosis, innate immune signaling, chronic inflammation, and systemic autoinflammatory diseases. However, the role and mechanism of DNASE2's action in gliomas remain unclear. In this study, the difference analysis showed that after supplementing normal tissue samples from the Genotype-Tissue Expression (GTEx) dataset, DNASE2 mRNA levels in 30 tumors from The Cancer Genome Atlas (TCGA) showed significant differences, correlated with a poor prognosis in patients with glioblastoma multiforme (GBM). DNASE2 down-regulation also reduced GBM cell proliferation, migration, and invasion. DNASE2 affected the immune activity of GBM cells. Furthermore, we found that interleukin-17, Toll-like receptor, North signaling pathway, secreted phosphoprotein 1 (SPP1), and S100 calcium-binding protein A8/9 (S100A8/9) genes may be crucial to regulate GBM immunity via DNASE2. In conclusion, DNASE2 expression is elevated in patients with GBM and influences the development of GBM, possibly through various immune-related pathways. Therefore, DNASE2 may serve as a potential prognostic biomarker for GBM. The overall workflow of this study is shown in Figure 1A.

**Figure 1:**
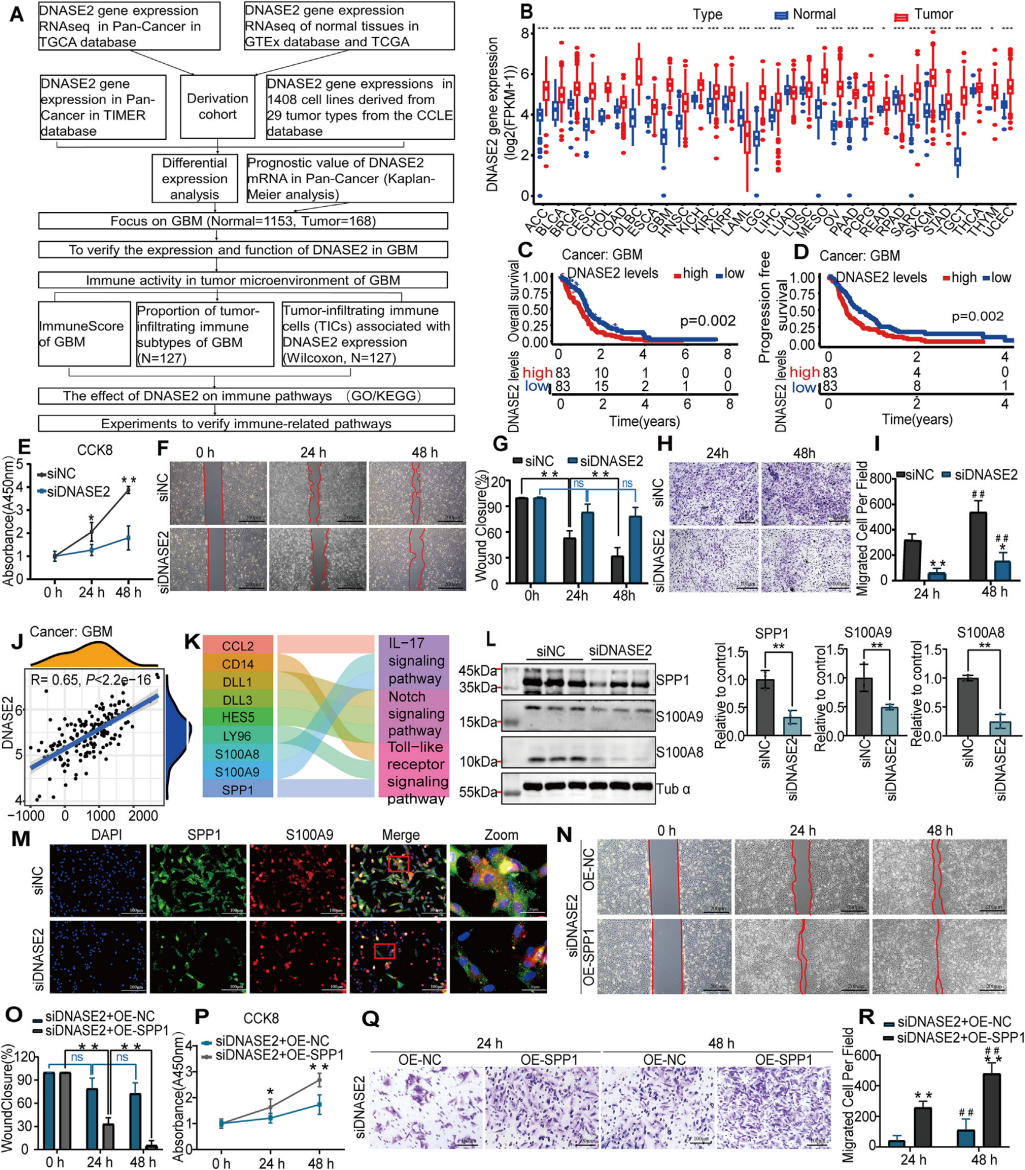
DNASE2 mRNA profile, prognostic value in pan-cancer, and key role in immune activity in the tumor microenvironment of glioblastoma multiforme (GBM). **(A)** Workflow of this study. **(B)** DNASE2 was differentially expressed among multiple cancer categories and normal tissues according to the TCGA and GTEx databases. Individual boxplots represent DNASE2 content (Illumina HTSeq-FPKM: log2 (FPKM+1)) in various cancer forms. Red and blue indicate cancer and normal tissues, respectively. ∗*p* < 0.05, ∗∗*p* < 0.01, and ∗∗∗*p* < 0.001, based on the Wilcoxon test. **(C, D)** Kaplan–Meier analysis of the correlation between DNASE2 expression and overall survival (OS) (C) and progression-free survival (PFS) (D) of patients with GBM (*p* < 0.05). **(E**) The proliferation ability of U251 cells after transfection with siNC and siDNASE2 was verified using the cell counting kit-8 (CCK8) assay. ∗*p* < 0.05 and ∗∗*p* < 0.01 versus the negative control (NC) group. **(F, G)** The migration ability of U251 cells after transfection with siNC and siDNASE2 was verified using a wound healing assay. ∗∗*p* < 0.01. **(H, I)** The invasion ability of U251 cells after transfection with siNC and siDNASE2 was verified using a Transwell assay. ∗*p* < 0.05 and ∗∗*p* < 0.01 versus the NC group; ^##^*p* < 0.01 versus the 24 h group. **(J)** The scatter plots of the correlation between ImmuneScore and DNASE2 expression in GBM (*p* < 0.05). The blue lines within the plots represent the fitted linear model that indicates the proportional tropism of ImmuneScore and DNASE2 expression. The Spearman's test assessed the correlation significance. **(K)** The genes enriched in KEGG pathways. **(L)** Protein expression levels of SPP1, S100A8, and S100A9 in U251 cells after transfection with siNC and siDNASE2 were examined by western blotting. ∗∗*p* < 0.01. **(M)** Protein profiles of SPP1 and S100A9 in U251 cells after transfection with siRNA negative control (siNC) and DNASE2 siRNA (siDNASE2) were examined by immunofluorescence 24 h after siRNA transfection. **(N, O)** U251 cell migratory ability following transfection with siDNASE2+OE-NC and siDNASE2+OE-SPP1 was verified using a wound healing assay. ∗∗*p* < 0.01. **(P)** U251 cell proliferative ability following transfection with siDNASE2+OE-NC and siDNASE2+OE-SPP1 was verified using the CCK8 assay. ∗*p* < 0.05 and ∗∗*p* < 0.01 versus the siDNASE2+OE-NC group. **(Q, R**) U251 cell invasive ability following transfection with siDNASE2+OE-NC and siDNASE2+OE-SPP1 was verified using a Transwell assay. ∗∗*p* < 0.01 versus the siDNASE2+OE-NC group; ^##^*p* < 0.01 versus the 24 h group. Data were displayed as mean ± standard deviation.

DNASE2 belongs to the DNA-encoding family of enzymes located in lysosomes and hydrolyzes DNA under acidic conditions.^1^ DNASE2 regulates cell proliferation, migration, invasion, apoptosis, and other processes through various signaling pathways.^2^ However, studies on DNASE2 expression in malignant tumors are limited. Herein, we obtained gene expression profile data, which included 10,191 tumors and 730 normal tissues from 31 cancer categories in the TCGA database and 7862 normal tissues in the GTEx database of UCSC Xena (https://xena.ucsc.edu/), and assessed DNASE2 mRNA levels between human tumors and normal tissues. For tumors without normal tissue samples from the TCGA database, we supplemented the corresponding samples from the GTEx database and corrected them to ensure data comparability. Difference analysis showed that DNASE2 mRNA levels increased strongly in 27 tumors (*p* < 0.05) and decreased significantly in three tumors (*p* < 0.05). No significant differences were observed in lung squamous cell carcinoma (Fig. 1B). We then sequenced 31 tumor tissues using mRNA expression levels of DNASE2. The median expression level of DNASE2 was the lowest in acute myeloid leukemia (3.02) and highest in mesothelioma (5.91) (Fig. S1A).

mRNA expression levels in tumor tissues may be affected by tumor-adjacent non-tumor tissues, we downloaded DNASE2 transcript expression in 1408 cell lines from 29 tumor types based on the Cancer Cell Line Encyclopedia (https://portals.broadinstitute.org/ccle/) database. Cell lines from the adrenal gland, skin, central neural system/brain, prostate, and eye expressed the highest amount of DNASE2 transcripts, while cell lines from the testis, vulva/vagina, peripheral nervous system, myeloid, and lymphoid tissues expressed relatively lower levels of DNASE2 mRNA (Fig. S1B).

We then downloaded all data related to tumor survival to extensively assess the relationship between DNASE2 mRNA expression and pan-cancer prognosis. Kaplan–Meier analysis revealed correlations between DNASE2 mRNA expression and overall survival and progression-free survival (Fig. S1C, D). As a result, DNASE2 mRNA expression was correlated with overall survival and progression-free survival only in GBM (Fig. 1C, D). Furthermore, the DNASE2 content in GBM tumor tissues and tumor cell lines was among the top five in pan-cancer; therefore, GBM tumors were selected for further studies.

The results of the Gene Expression Omnibus (GEO) database revealed that, relative to the normal group, DNASE2 expression in the GBM tumor group increased significantly (*p* < 0.05) (Fig. S2A, B). We then explored DNASE2 protein profiles using the Human Protein Atlas database (https://www.proteinatlas.org/). Immunohistochemical staining suggested that DNASE2 was barely expressed in normal brain tissues. Conversely, moderate levels were observed in glioma tissues (Fig. S2C).

Subsequently, we explored the effect of DNASE2 on basic GBM cell function through a loss of function assay using siDNASE2 or siNC transfected into the human GBM cell line U251. Western blot analysis showed that siDNASE2-01 and siDNASE2-02 significantly reduced DNASE2 protein levels compared with the siNC group, and siDNASE2-01 was more effective in reducing DNASE2 expression than siDNASE2-02 (Fig. S2D). Therefore, siDNASE2-01 was used for further experiments.

We explored the effects of DNASE2 knockdown on the proliferation, migration, and invasiveness of U251 cells. The proliferation capacity of siDNASE2-transfected cells at 24 h and 48 h was reduced compared with that of the siNC group at the corresponding transfection times (Fig. 1E). Similarly, siDNASE2-transfected cell migration and invasion abilities at 24 h and 48 h were significantly lower than those of the siNC group (Fig. 1F–I). We also verified the function of DNase2 in the proliferation and invasion of U87 cells, and the results were consistent with those of the U251 cell lines (Fig. S2E, F).

The tumor microenvironment is a complex ecosystem that plays a crucial role in malignant tumor progression,^3^ immune escape,^4^ and treatment resistance.^5^ Therefore, the development of safe and effective immunotherapies based on the characteristics of tumor microenvironment is essential. The immune score for each tumor sample was calculated (Table S1). There was a positive correlation between the DNASE2 mRNA profile and the GBM ImmuneScore (Spearman's correlation coefficient = 0.65; *p* = 2.2e−16; Fig. 1J). In the TCGA database, except for GBM samples with *p* > 0.05, 127 GBM cancer samples were selected for further study. Twenty-two immune cell distributions were constructed in GBM and the proportions of tumor-infiltrating immune subcategories were analyzed based on GBM samples (Fig. S3A). All 127 GBM samples in the TCGA database were separated into DNASE2 elevated and reduced expression groups using the median DNASE2 transcript quantity as the cut-off value. The Wilcoxon rank-sum test was used to analyze changes in tumor-infiltrating immune cells (TICs) in the two groups to elucidate which types of tumor-infiltrating immune cells were related to the DNASE2 profile. DNASE2 mRNA expression was positively correlated with resting memory CD4^+^ T cells and activated dendritic cells, while negatively correlated with four types of tumor-infiltrating immune cells including naïve B cells, follicular helper T cells, resting natural killer cells, and M2 macrophages (Fig. S3B, C). We collected tissue samples from six patients with GBM and detected the expression levels of DNASE2 using quantitative reverse-transcription PCR (Fig. S3D). We divided the six samples into the DNASE2 high and DNASE2 low groups. The expression levels of CD20, CD3, and CD68 were detected by immunohistochemistry. CD20 is a B-lymphocyte marker, CD3 is a T-lymphocyte marker, and CD68 is a mononuclear macrophage marker. The results showed that, compared with the DNASE2 low group, CD3 expression increased in the DNASE2 high group and CD68 expression decreased (Fig. S3E, F). These results were consistent with resting memory CD4^+^ T cells and M2 macrophages shown in Figure S3C. Based on this evidence, the DNASE2 profile moderately modulates tumor microenvironment-based immune activity.

To investigate the effect of DNASE2 on immune pathways, we downloaded the transcriptome data of patients with GBM from the TCGA database and separated the samples into DNASE2 elevated- and reduced-content groups using the DNASE2 median as the cut-off value. Differential gene expression analysis between the two groups and Gene Ontology (GO) enrichment analysis were performed (Fig. S4A, B). Kyoto Encyclopedia of Genes and Genomes (KEGG)–enriched pathways that include interleukin-17, Toll-like receptor, and Notch signaling pathways (Fig. S4C). Genes enriched in these pathways were closely related to immunity (Fig. 1K).

Furthermore, we focused on the immune pathway proteins SPP1 and S100A8/9, which were strongly up-regulated in the DNASE2 elevated expression group and detected siDNASE2-mediated regulation of SPP1 and S100A8/9 expression levels. Immunofluorescence and Western blot analyses revealed that SPP1 and S100A8/9 expression levels in the siDNASE2 group were markedly lower than those in the siNC group (Fig. 1L, M). We examined the effects of SPP1 overexpression on U251 cell proliferation, migration, and invasion. The proliferative capacity of siDNASE2-transfected cells was reversed by OE-SPP1 transfection. At 24 h and 48 h, the proliferative capacity of U251 cells increased compared with that of the siDNASE2+OE-NC group at the corresponding transfection times (Fig. 1P). Similarly, the migration and invasion abilities of the siDNASE2+OE-SPP1 group at 24 h and 48 h were significantly higher than those of the siDNASE2+OE-NC group at the corresponding transfection times (Fig. 1N, O, Q, R).

In summary, this study used DNASE2 as a potential target for GBM and explored DNASE2 expression in pan-cancer and prognostic analyses, focusing on the critical role of DNASE2 in the GBM tumor microenvironment for the first time and providing a new dimension for future research on GBM. However, this study had some limitations. First, due to the source of the data, we combined GTEx and TCGA database genetic data; therefore, there may be some inter-batch differences. Second, the verification information for DNASE2 in our experimental data was insufficient.

## Funding

This work was funded by the 10.13039/501100001809National Natural Science Foundation of China (No. 82373403 to Y.C.) and the Beijing New-Star Plan of Science and Technology (China) (No. Z201100006820148 to Y.C.).

## CRediT authorship contribution statement

**Jun Cao:** Investigation. **Si Chen:** Investigation, Methodology. **Ping An:** Data curation, Investigation. **Xingqiang Wang:** Investigation, Software, Validation. **Xinru Xiao:** Formal analysis, Supervision, Validation. **Shichao Li:** Writing – original draft. **Ye Cheng:** Writing – review & editing.

## Conflict of interests

There is no conflict of interests to declare.

## Figures and Tables

**figs1:**
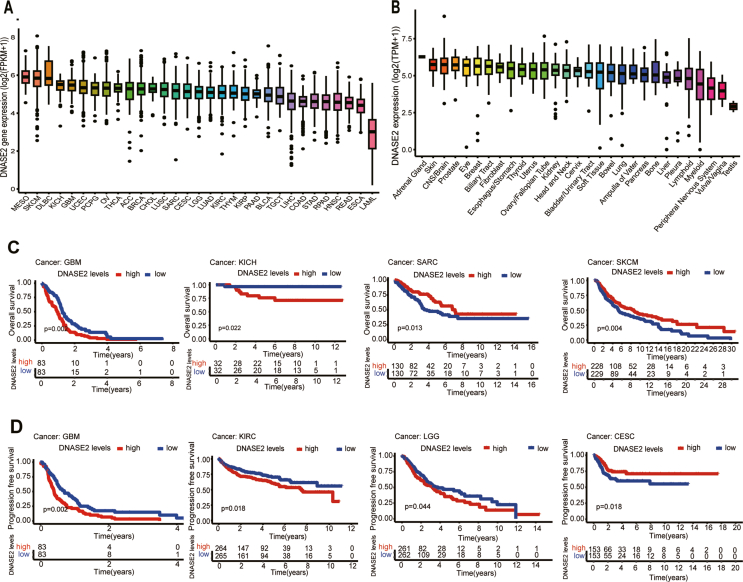


**figs2:**
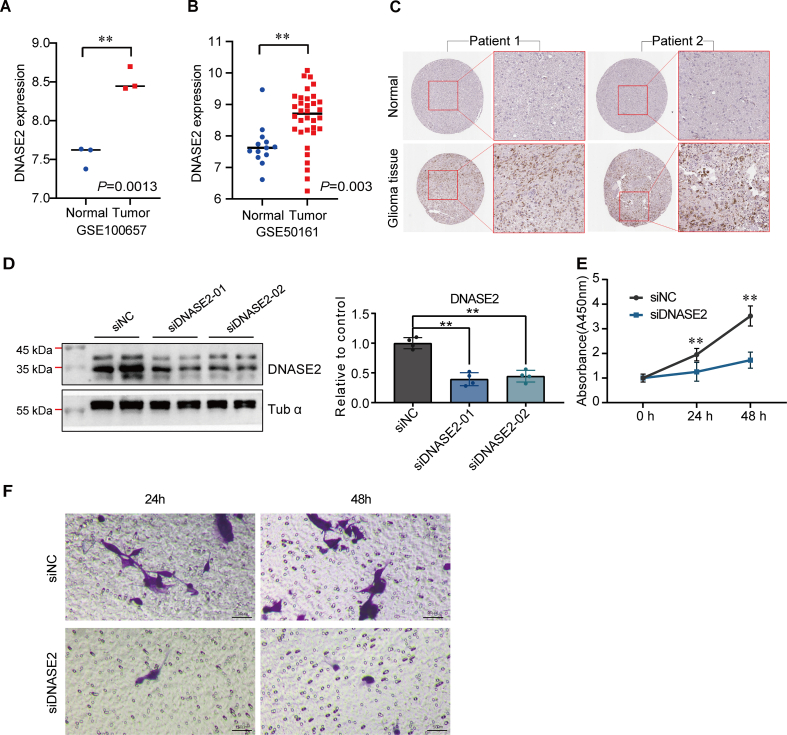


**figs3:**
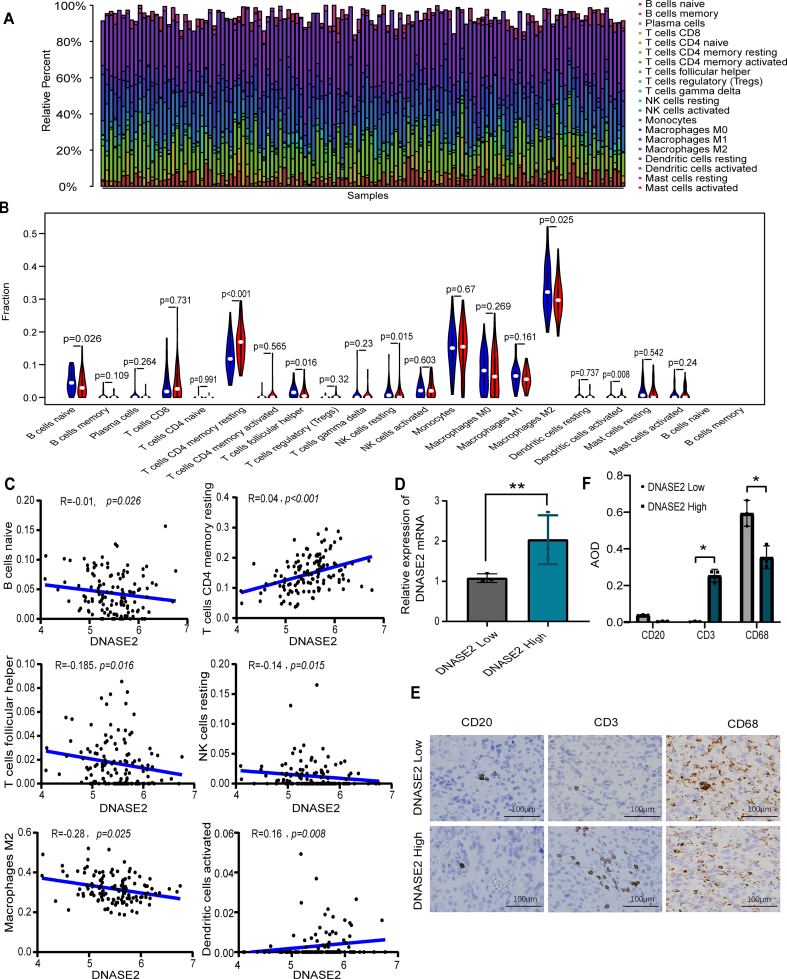


**figs4:**