# Supplementary material for: Systematic pan-cancer analysis identifies DNASE2 as a potential prognostic marker and immunotherapeutic target for glioblastoma multiforme
Source: Genes Dis. 2024 Sep 10;12(4):101431. doi: 10.1016/j.gendis.2024.101431 (PMC11984583; doi:10.1016/j.gendis.2024.101431)
Supplement: Multimedia component 1 [file mmc1.docx]

**Systematic Pan-Cancer Analysis Identifies DNASE2 as a Potential Prognostic Marker and Immunotherapeutic Target for Glioblastoma Multiforme**

**Running title:** DNASE2 is a prognostic marker to target glioblastoma multiforme

**Materials and** **methods**

**TCGA and GTEx datasets download**

The gene expression profile data, which included 10191 tumor and 730 normal tissues from 31 cancer categories in the TCGA database and 7862 normal tissues in the GTEx database, were obtained from UCSC Xena (<https://xena.ucsc.edu/>). These data were used as validation datasets to establish a prognostic model. The ‘sva’ package in R software was employed for batch-normalization of gene expression from various databases ^1, 2^.

**Analyses of DNASE2 mRNA expressions in pan-cancer and normal tissues**

Cancer categories with at least two normal tissues were assessed and the Wilcoxon test was used for the differential expression of DNASE2 mRNA between tumor and normal tissues. The aforementioned information was graphed using the ggpubr package in the R software. The tumor groups were arranged in descending order based on the median value of DNASE2 transcript levels in pan-cancer and visualized using the plyr and ggpubr packages (R). Statistically significance was set at *p* < 0.05.

**DNASE2 mRNA content in cancer cell lines**

DNASE2 RNA sequencing (RNA-seq) information for human cancer cell lines (CCLs) were acquired from the Cancer Cell Line Encyclopedia (CCLE, <https://portals.broadinstitute.org/ccle/)> database, which included DNASE2 mRNA expression. The DNASE2 RNA-seq data contained 1408 human CCLs. Human CCLs were arranged in descending order based on the median DNASE2 content in each cell line and visualized via plyr and ggpubr packages (R).

**DNASE2 mRNA prognostic significance in pan-cancer**

Survival and DNASE2 expression data for pan-cancer were obtained from the TCGA database. Kaplan–Meier (K-M) assessment was conducted to examine the correlation between DNASE2 levels and patients’ overall survival (OS) and progression-free survival (PFS) in pan-cancer. Subjects with various cancer types were separated into elevated and reduced DNASE2 content groups using the median DNASE2 content as the cut-off value. Survival curves were constructed using K-M analysis ^3^. Statistical assessments were conducted via the survival package, and visualization achieved via the survminer and forestplot packages. Statistically significance was set at *p* < 0.05.

**Verification of DNASE2 mRNA expression in GBM**

GSE100657 and GSE50161 from the Gene Expression Omnibus (GEO) database were employed for the DNASE2 transcript level alteration verification between GBM and normal tissues. R software v. x64 4.1.2 was used for statistical analysis, and the limma and beeswarm packages were employed for variance assessment and scatter plots, respectively. Inter-group differences were assessed via the Wilcoxon test. Statistically significance was set at *p* < 0.05.

**Determination of the ImmuneScore, StromalScore, and ESTIMATEScore**

The ESTIMATE package (R, v. x64 4.1.2) ^4^ was employed for the determination of the ImmuneScore (immune composition), StromalScore (stromal composition), and ESTIMATEScore (summation of the ImmuneScore and StromalScore) for individual tumor samples, where a higher score represents a larger proportion of that component (immune, stromal, and tumor purity) in the TME.

**Link between DNASE2 content and immune cell infiltration pattern in GBM**

GBM RNA expression data were acquired from the TCGA database. CIBERSORT was used to evaluate the proportion of tumor-infiltrating immune cells (TICs) profile in GBM cases. Cases with *P* < 0.05 were selected for the follow-up analyses. GBM samples were separated into DNASE2 elevated and reduced-content groups using the median DNASE2 content as the cut-off value. Inter-group TIC expression assessment utilized the Wilcoxon test, and visualization was completed using the vioplot package ^5^. Finally, the DNASE2 content and tumor-infiltrating immune cells link was assessed via Spearman’s correlation analysis using IBM SPSS software. Statistically significance was set at *p* < 0.05.

**Gene Ontology (GO) and Kyoto Encyclopedia of Genes and Genomes (KEGG) enrichment assessments**

GO and KEGG enrichment assessments were conducted on genes that differed significantly between the DNASE2 elevated and reduced-content groups. Statistical analyses utilized the clusterProfiler package, and visualization was achieved with the ggplot2 package ^6, 7^. Statistically significance was set at *p* < 0.05.

**Cell culture and transfection**

The GBM cell line U251 (CL-0237, Pricella) and U87 (ATCC lot number: 63710285) were grown in Dulbecco’s modified Eagle’s medium (C11995500BT, Gibco, Waltham, MA, USA) with 10% fetal bovine serum (FBS) (Gibco) and 1% antibiotics (penicillin and streptomycin) at 37°C in 5% CO_2_. For DNASE2 knockdown, DNASE2 siRNA (siDNASE2, Ribobio Co., Ltd., Guangzhou, China) was transfected using the Lipo3000 transfection reagent (Thermo Fisher Scientific, Waltham, MA, USA) according to the manufacturer’s instruction. The employed DNASE2 siRNA sequences were #1: CAAGAACCCUGGAACAGCAGCAUCA and #2: GCCTTCTCTTCCCTCTCTCC. Knockdown efficiency was confirmed using western blotting as previously described ^8^.

**Western blotting**

Cellular proteins were electrophoresed in sodium dodecyl sulfate-polyacrylamide gel, prior to transfer to polyvinylidene difluoride membranes. The primary antibodies used for immune incubation were anti-DNASE2 (1:1000, ab8119, Abcam, Waltham, MA, USA), anti-SPP1 (1:2000, 22952-1-AP, Proteintech, China), anti-S100A8 (1:600,15792-1-AP, Proteintech), and anti-S100A9 (1:1000, MA1-81381, Invitrogen, Waltham, MA, USA). Following an overnight exposure to primary antibodies at 4°C, membranes were exposed to goat anti-rabbit or anti-mouse secondary antibodies (1:5000, 074-1506, 074-1806; Kirkegaard & Perry Laboratories, Gaithersburg, MD, USA) at room temperature for 1 h. Bound antibodys visualization utilized ECL Prime Western blot Detection Reagent (ZD310A, ZomanBio, Beijing, China). The resulting data was normalized to the loading control (Tub α, 1:5000, 60004-1-Ig, Proteintech, China) and expressed as fold change of specific bands versus the control group.

**qRT-PCR**

Total RNA was extracted using TRIzol reagent (Life Technologies Corporation, Carlsbad, CA, USA). RNA concentrations were detected and the quality was determined at 260/280 nm absorbance using a microplate reader (BioTek, USA). ChamQ Universal SYBR qRCR Master Mix (Vazyme Biotech Co., Ltd., Nanjing, China) was applied to determine mRNAs expressions of DNASE2 and GAPDH. The fold change was equivalent to the relative expression normalized to endogenous control (2^−ΔΔCt^). GAPDH was used as internal control. The primer sequences of DNASE2 were Forward Primer: GCCAGCTCTTAGAGGGTCC and Reverse Primer: CTCCGGGCTGTTGATGAGT. The primer sequences of GAPDH were Forward Primer: GCCAAAAGGGTCATCATCTCTG and Reverse Primer: CATGCCAGTGAGCTTCCCGT.

**Immunofluorescence**

U251 cells transfected with siRNA negative control (siNC) or siDNASE2 were seeded onto the chamber slides. Immunostaining was performed after fixing with 4% paraformaldehyde for 30 min. Slides underwent a 15-min exposure to QuickBlock™ immunostaining blocking solution (P0260, Beyotime, Shanghai, China) for nonspecific antigens blocking and were then incubated with anti-SPP1 (1:150, 22952-1-AP, Proteintech) and anti-S100A9 (1:100, MA1-81381, Invitrogen) overnight at 4°C. The following day, the slides were exposure to Alexa Fluor 488 goat anti-rabbit IgG(H+L) and Alexa Fluor 555 donkey anti-mouse IgG(H+L) secondary antibodies (A0423, A0453, Beyotime, Shanghai, China) at 37°C for 1 h. Following sealing with an anti-fluorescence quenching sealing solution supplemented with DAPI (P0131; Beyotime, Shanghai, China), evaluation was completed under a fluorescence microscope (Nikon, ECLIPSE Ti, Japan).

**Hematoxylin-eosin (H&E) and immunohistochemistry (IHC) staining**

H&E staining were performed as a standard protocol. Briefly, incubating hematoxylin, rinsing, incubating eosin, then dehydration, clarification and coverslipped. IHC staining were performed on 5μm sections using standard methods. Sections were rehydrated, quenching endogenous peroxidase and blocking endogenous biotin and non-specific interactions and then incubated with primary antibodies including CD3 (ZA0503, ZSGB-BIO, Beijing, CHINA), CD20(ZM-0039, ZSGB-BIO, Beijing, CHINA) and CD68 (ZM0060, ZSGB-BIO, Beijing, CHINA). Biotinylated secondary antibodies for imaging. Digital photography was performed by Olympus VS120 Virtual Slide Microscope (Olympus, VS120-S6-W, Japan). The analysis of IHC imaging was performed by ImageJ software, the background of every sample was subtracted.

**Cell counting kit-8 (CCK8) assessment**

The U251 cells and U87 cells transfected with siNC or siDNASE2 were seeded into 96-well plates at a density of 1×10^4^ cells/well. CCK8 reagent (10 μL/well; Dojindo Molecular Technologies, Dojindo, Japan) solution was added at 0, 24, and 48 h of cell culture following the manufacturer’s instructions. Optical density was determined at 450 nm following incubation at 37°C in 5% CO_2_ for 30 min.

**Wound healing**

U251 cells transfected with siNC or siDNASE2 were seeded in a 6-well plate at a density of 5×10^5^ cells/well. When cells reached the sub fusion state, the culture wells were crossed vertically with a pipette tip. The culture supernatant was aspirated, and the cells were rinsed in PBS 2–3 times to eliminate detached cells. Serum medium (1%; 2 mL) was introduced to individual wells, followed by incubation at 37°C in 5% CO_2_. Images were acquired at 0, 24, and 48 h of cell culture, and the wound area was measured using the Fiji software.

**Transwell assay**

Matrigel (356234, Corning, NY, USA) was diluted with culture medium at a ratio of 1:8 on ice. The U251 cells or U87 cells transfected with siNC and siDNASE2 (1×10^4^ cells/well) were seeded in the top chambers of the Transwell plates (8 μm pore size, Corning) in FBS-free media with Matrigel-coated membrane inserts. The lower chamber was filled with a medium containing 10% FBS. After 24 h of incubation, cells were fixed with 4% paraformaldehyde for 30min and stained with 0.1% crystal violet for 20 min.

**Statistical analysis**

All bioinformatics analyses carried out in this study were performed using R 4.3.1 (unless otherwise stated). Statistical analyses of experimental data were conducted via SPSS software (version 22.0; IBM Corp., Armonk, NY, USA). All results are expressed as the mean ± SD. Inter-group assessments utilized the t-test, and multiple-group comparisons were performed using one-way ANOVA followed by the LSD test. Significance was set at *p* < 0.05.

**References**

1. Leek J T, Johnson W E, Parker H S, et al. The sva package for removing batch effects and other unwanted variation in high-throughput experiments. *Bioinformatics (Oxford, England).* 2012;28(6):882-3. doi.org/10.1093/bioinformatics/bts034.

2. Wei S, Teng S, Yao J, et al. Develop a circular RNA-related regulatory network associated with prognosis of gastric cancer. *Cancer medicine.* 2020;9(22):8589-8599. doi.org/10.1002/cam4.3035.

3. Zhang C, Guo C, Li Y, et al. Identification of Claudin-6 as a Molecular Biomarker in Pan-Cancer Through Multiple Omics Integrative Analysis. *Front Cell Dev Biol.* 2021;9:726656. doi.org/10.3389/fcell.2021.726656.

4. Yoshihara K, Shahmoradgoli M, Martínez E, et al. Inferring tumour purity and stromal and immune cell admixture from expression data. *Nat Commun.* 2013;4:2612. doi.org/10.1038/ncomms3612.

5. Xu F, Shen J,Xu S. Integrated Bioinformatical Analysis Identifies GIMAP4 as an Immune-Related Prognostic Biomarker Associated With Remodeling in Cervical Cancer Tumor Microenvironment. *Front Cell Dev Biol.* 2021;9:637400. doi.org/10.3389/fcell.2021.637400.

6. Subramanian A, Tamayo P, Mootha V K, et al. Gene set enrichment analysis: a knowledge-based approach for interpreting genome-wide expression profiles. *Proc Natl Acad Sci U S A.* 2005;102(43):15545-50. doi.org/10.1073/pnas.0506580102.

7. Yu G, Wang L G, Han Y, et al. clusterProfiler: an R package for comparing biological themes among gene clusters. *Omics.* 2012;16(5):284-7. doi.org/10.1089/omi.2011.0118.

8. Takahashi A, Loo T M, Okada R, et al. Downregulation of cytoplasmic DNases is implicated in cytoplasmic DNA accumulation and SASP in senescent cells. *Nat Commun.* 2018;9(1):1249. doi.org/10.1038/s41467-018-03555-8.
