# Supplementary material for: Systematic pan-cancer analysis identifies DNASE2 as a potential prognostic marker and immunotherapeutic target for glioblastoma multiforme
Source: Genes Dis. 2024 Sep 10;12(4):101431. doi: 10.1016/j.gendis.2024.101431 (PMC11984583; doi:10.1016/j.gendis.2024.101431)
Supplement: Multimedia component 2 [file mmc2.docx]

**Systematic Pan-Cancer Analysis Identifies DNASE2 as a Potential Prognostic Marker and Immunotherapeutic Target for Glioblastoma Multiforme**

**Running title:** DNASE2 is a prognostic marker to target glioblastoma multiforme

**Table S1 Estimation for ImmuneScore, StromalScore, and ESTIMATEScore**

**
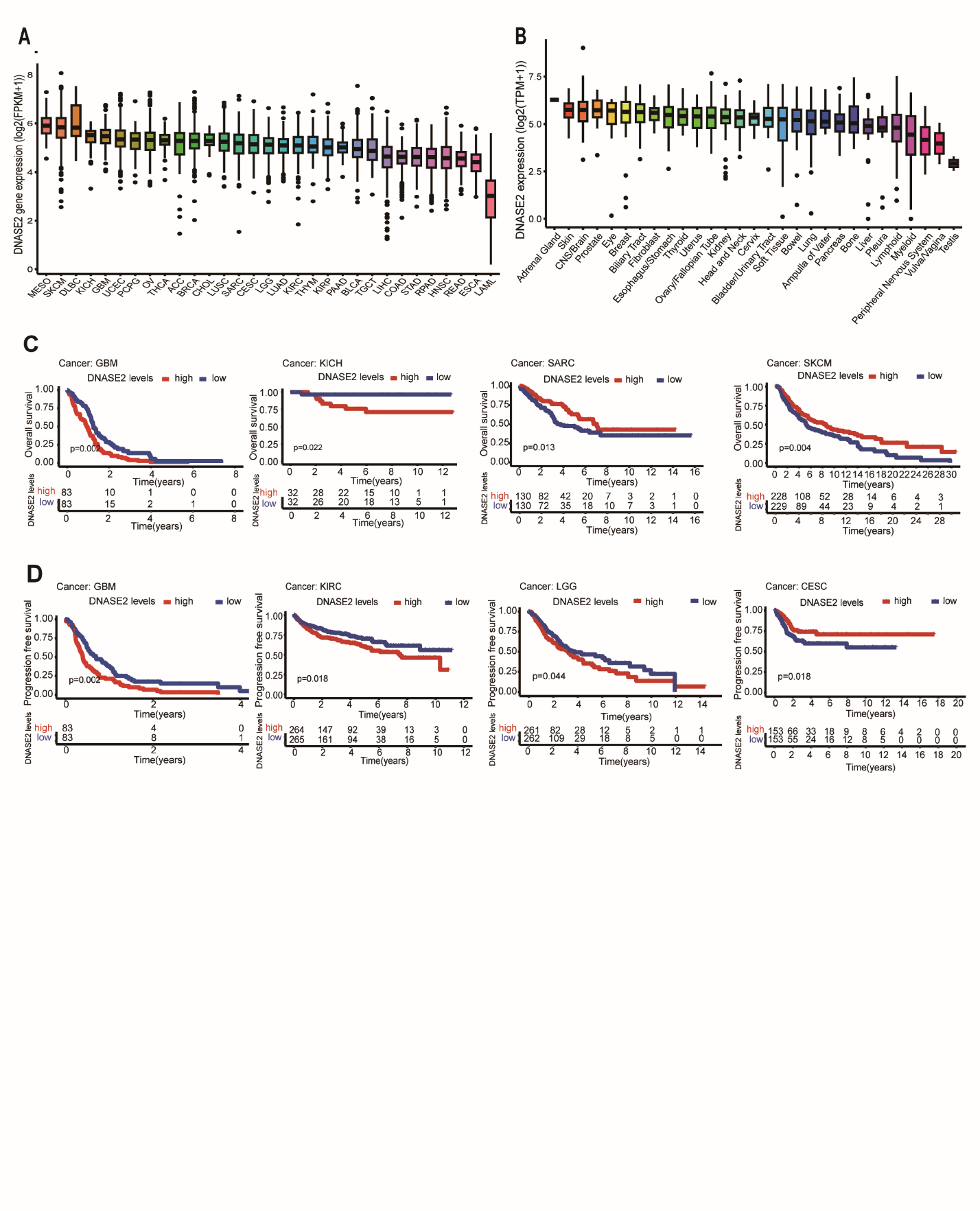
presented in Excell**

**Figure S1** DNASE2 mRNA profile and prognostic value in pan-cancer. (**A)** The pan-cancer types were arranged in decreasing order according to the median value of DNASE2 expression. The bar represents the median expression of cancers or normal tissues, whereas the bottom and top ends of the box represent the 25th and 75th percentile profiles, respectively. (**B)** DNASE2 contents in varying tumor cell lines is presented according to the CCLE database (https://portals.broadinstitute.org/ccle/). (**C–D)** Kaplan–Meier analysis of the correlation between DNASE2 expression and overall survival (OS) (C) and progression-free survival (PFS) (D) of patients in pan-cancer with *P* < 0.05.


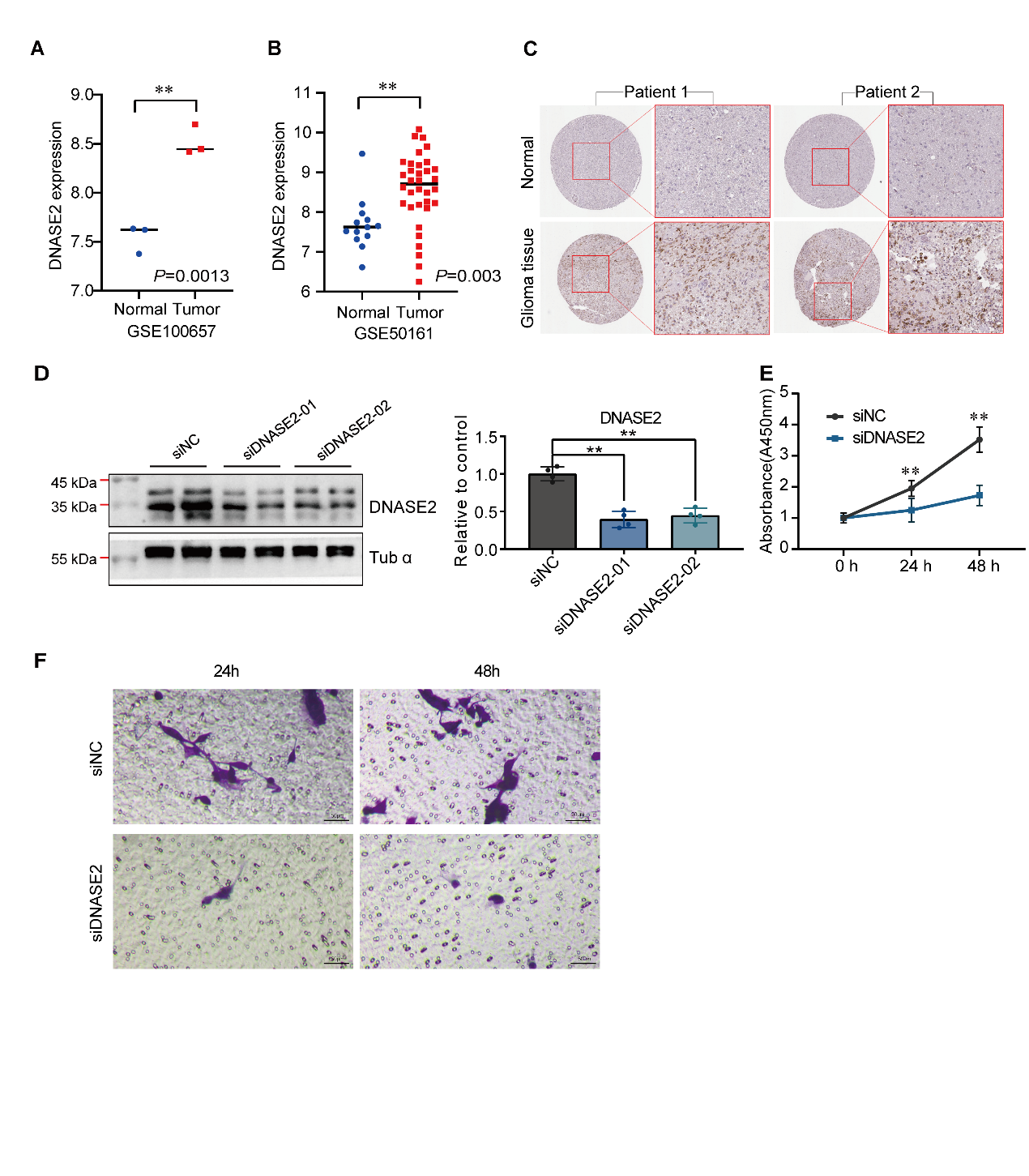
**Figure S2** Expression verification of DNASE2 and silencing effect of siDNASE2 in glioblastoma multiforme (GBM). (**A–B)** DNASE2 expression verification in GBM from the Gene Expression Omnibus (GEO) database. (**C**) DNASE2 protein levels in the HPA database. (**D)** The silencing effect of siDNASE2-01 and siDNASE2-02 was verified using western blotting. **(E)** The proliferation ability of U87 cells after transfection with siNC and siDNASE2 was verified using the cell counting kit-8 (CCK8) assay. ***P* < 0.01 compared with the negative control (NC) group. **(F)** The invasion ability of U87 cells after transfection with siNC and siDNASE2 was verified using a Transwell assay. Data are displayed as the mean ± SD. ***P* < 0.01 compared with the negative control (NC) group.


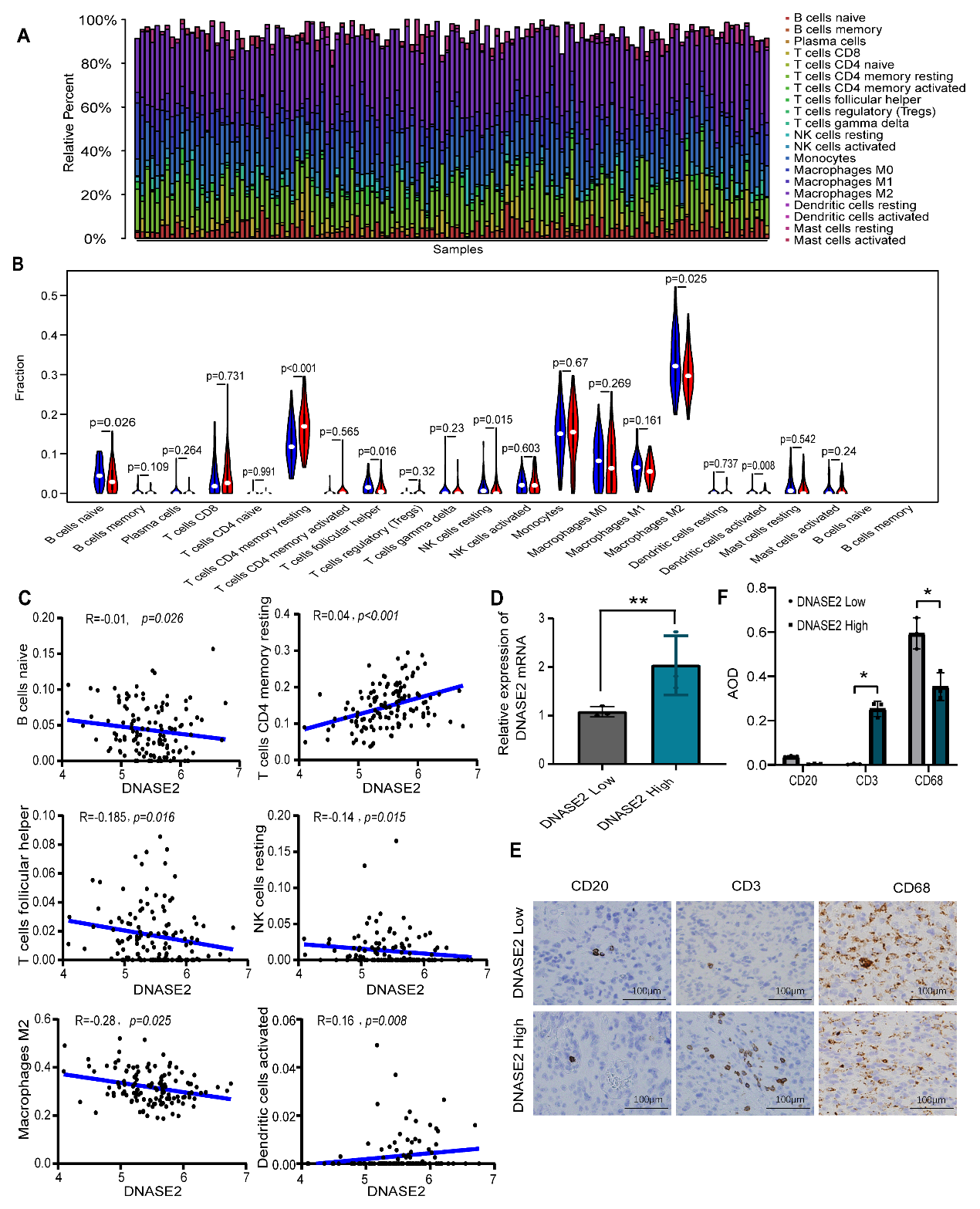


**Figure S3** Relationship between DNASE2 and the GBM microenvironment. **(A)** Barplot showing the distribution of 22 types of tumor-infiltrating immune cells (TICs) in GBM cancer samples. Column names represent sample IDs. (**B)** Violin plot depicting the distribution of 22 TICs forms between DNASE2 high- and low-expression groups in GBM. The Wilcoxon rank sum determined statistical significance. (**C)** The scatter plot shows the correlation of six types of TICs in proportion to DNASE2 content (*P* < 0.05). Blue lines within plots represent a fitted linear model that indicates the proportion tropism of the immune cell sand DNASE2 expression, and Spearman’s correlation analysis was used for the correlation test. **(D)** DNASE2 mRNA expression in tissues of 6 GBM patients. (**E and F**) Representative immunohistochemical staining photomicrographs show the expression of CD20, CD3 and CD68 in tissues of 6 GBM patients **(E)** and its AOD measured using ImageJ software **(F)** (scale bar = 100 μm). **P* < 0.05 and ***P* < 0.01. Data are displayed as the mean ± SD.


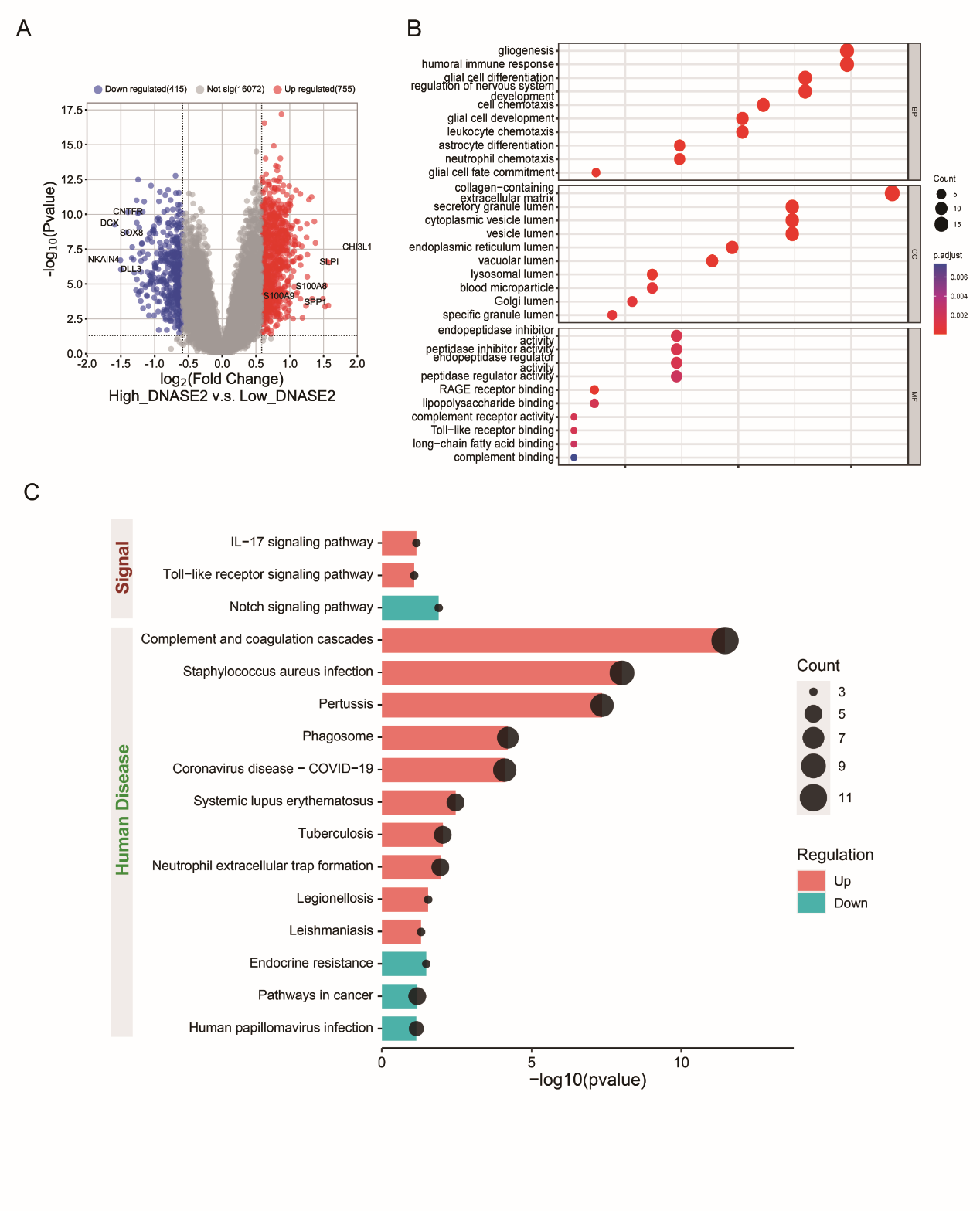


**Figure S4** Enrichment analyses of DNASE2. (**A)** Gene differential expression analysis between DNASE2 high- and low-expression groups based on the median DNASE2 expression of patients with GBM from the TCGA database. **(B)** Gene Ontology (GO) enrichment analysis. (**C)** Kyoto Encyclopedia of Genes and Genomes (KEGG) pathway enrichment analysis.
